# Evaluating the effects of hospital wastewater treatment on bacterial composition and antimicrobial resistome

**DOI:** 10.3389/fmicb.2025.1620677

**Published:** 2025-10-02

**Authors:** Yan Meng, Yi Xu, Dongping Hu, Qiuxiang Pan, Liangliang Weng, Weiyi Huang, Jin Zhao, Wei Lan, Qiucheng Shi, Yunsong Yu, Yan Jiang

**Affiliations:** ^1^Department of Clinical Laboratory, Zhejiang Hospital, Zhejiang University School of Medicine, Hangzhou, China; ^2^Department of Infectious Diseases, Sir Run Run Shaw Hospital, Zhejiang University School of Medicine, Hangzhou, China; ^3^Key Laboratory of Microbial Technology and Bioinformatics of Zhejiang Province, Hangzhou, China; ^4^Regional Medical Center for National Institute of Respiratory Diseases, Sir Run Run Shaw Hospital, Zhejiang University School of Medicine, Hangzhou, China; ^5^Department of Hospital Epidemiology and Infection Control, Sir Run Run Shaw Hospital, Zhejiang University School of Medicine, Hangzhou, China

**Keywords:** hospital wastewater treatment, antimicrobial resistance gene, metagenomic sequencing, mobile genetic elements, wastewater treatment effectiveness

## Abstract

Hospital wastewater treatment systems (HWTS) are crucial in reducing the spread of antimicrobial resistance genes (ARGs) and antibiotic-resistant bacterial pathogens in hospital wastewater. This study aims to evaluate the impact of HWTS on the changes of bacterial composition and the relative abundance of ARGs. We collected wastewater samples from influent and effluent in a university hospital, and performed metagenomic sequencing. The results showed that HWTS altered the bacterial composition, with a decrease in the proportion of *Gammaproteobacteria*. However, phylogenetic analysis of metagenome-assembled genomes showed that *Mycobacterium* and *Zoogloea* from influent and effluent had a close relationship. A total of 140 non-redundant ARGs were identified based on open reading fragments analysis, with beta-lactam and aminoglycoside resistance genes being the most prevalent. The relative abundance of ARGs generally decreased after wastewater treatment (*p* < 0.0001), with 70.0% of genes that conferring resistance to “last-resort” antibiotics being undetectable in the effluent. However, the relative abundance of quaternary ammonium compounds resistance genes increased in the effluent. We identified that 66.4% of ARGs were located on plasmids, and 17.9% of ARGs were adjacent to mobile gene elements (MGEs), suggesting their potential for mobility. Subsequent analysis showed that ARGs originating from plasmids and adjacent to MGEs were negatively associated with their relative abundance reduction. In conclusion, this study provides a comprehensive evaluation of the impact of HWTS on composition of bacteria and the relative abundance of ARGs, highlighting the importance of effective wastewater treatment in combating the spread of antimicrobial resistance.

## Introduction

Antimicrobial resistance (AMR) has emerged as a public issue of global concern (Velazquez-Meza et al., 2022; Salam et al., 2023). The current situation indicates that AMR is responsible for approximately 700,000 deaths annually, and forecasts predict that AMR could lead to 10 million deaths per year, and 100 trillion dollars in economic losses by 2050 (O’Neill, 2016). Moreover, this issue not only has an impact on human health, but also poses a major threat to the ecosystem. AMR negatively impacts biodiversity, and the presence of antimicrobial residues in the environment promotes the development of antibiotic-resistant bacteria (ARB). Therefore, effective control of AMR spreading is essential and urgently needed.

Except for the strict implementation of antimicrobial stewardship, controlling of antimicrobial resistance genes (ARGs) needs multifaceted efforts worldwide, which is the major concept of “One Health” (Zinsstag et al., 2011). Healthcare facilities, such as hospitals, play an important role in the “One Health” approach. Hospitals require large amounts of water for their proper operation, and generate large volume of wastewater (Wen et al., 2004). Hospital wastewater (HWW) is characterized by the presence of various emerging contaminants, including antibiotic residues and ARBs (Zhang et al., 2020). Hospital wastewater effluents are usually discharged into the city sewer system, however, most of these plants are not designed to effectively manage biomedical wastes. Thus, HWW is the significant sources of released ARGs into the municipal environment (Kang et al., 2024), which makes the HWW treatment within hospital facilities particularly important (Majumder et al., 2021).

Huang et al. (2023) reported that the profiles of ARGs within HWW were diverse influenced by geographical location and hospital type. Aminoglycoside, beta-lactam, macrolide, sulfonamide, and tetracycline resistance genes were predominant categories of ARGs, and *Pseudomonadota* emerged as the principal ARG carrier in the HWW at the phylum level (Huang et al., 2023). Previous study has revealed that mobile gene elements (MGEs) are well-documented vectors for the transmission of ARGs (Ellabaan et al., 2021), and ARGs-harboring plasmids had a positive correlation with the prevalence of ARGs in the HWW (Huang et al., 2023; Zhu et al., 2023).

To have a comprehensive understanding of the changes in bacterial composition and ARGs abundance after wastewater treatment in Sir Run Run Shaw Hospital, we collected samples of the influent and effluent from the hospital wastewater treatment system (HWTS), and performed metagenomic sequencing. Then, the bacterial taxonomy composition and variation were determined, and the phylogenetic relationship of metagenome-assembled genomes between influent and effluent was analyzed. Moreover, the changes of ARGs relative abundance were evaluated between influent and effluent to assess the effectiveness of the HWW treatment. Overall, this study would enhance our understanding of the impact of HWW treatment on bacterial composition and ARGs abundance, thereby contributing to the advancement of HWW treatment process.

## Materials and methods

### Sample origin and collection

Hospital wastewater samples were collected in September, 2022 at a tertiary hospital, Sir Run Run Shaw Hospital (Hangzhou, Eastern China) under non-rainy conditions. The HWTS is a three-stage process, and processes approximately 10,000 tons of wastewater per week. The primary treatment consists of a screen and a regulation tank, the secondary treatment involves a biological contact oxidation tank, and the tertiary treatment includes a disinfection tank, where 10% NaClO is added with the dosage automatically controlled based on the residual chlorine in the effluent. After treatment, the wastewater is discharged to a municipal wastewater treatment plant.

Wastewater samples (500 mL) were collected through single-time sampling, with one sample from the influent and one sample from effluent, using sterile waster bags. The samples were transported to laboratory immediately at 4 °C. A 50 mL subset of each sample was then filtered using the FluidPrep^™^ Concentrating Pipette System (InnovaPrep, Drexel, United States) with 0.1 μm PES Flat Filter Concentrating Pipette Tips, and the filter membranes were stored at −80 °C.

### Metagenomic sequencing, metagenomic assembly and open reading fragments prediction

DNA was extracted from sheared filter membranes using a DNeasy PowerSoil Pro Kit (QIAGEN, Hilden, Germany), and the concentration and purity of DNA was determined by Nanodrop 2000 (Supplementary Table S1, Thermo Scientific, Waltham, United States). Sequencing libraries were generated using the TIANSeq Fragment/Repair/Tailing module and TIANSeq Fast Ligation module (TIANGEN, Beijing, China) following the manufacturer’s recommendations. The kits employed a premixed enzyme module to fragment the DNA and simultaneously adds adapter. The prepared library was sequenced on Illumina NovaSeq (Illumina, San Diego, United States) with 150-bp paired-end strategy (Lan et al., 2023).

The raw reads were curated to obtain clean reads using SOAPnuke v1.5.0 (-l 15 -q 0.3 -n 0.05, Supplementary Table S2), and the clean reads were assembled using Megahit with K-mers ‘-k-list 45, 55, 67, 73’ (Li et al., 2015). The assembled scaffolds were broken from N linkage to obtain scaftigs (i.e., continuous sequences within scaffolds). The scaftigs shorter than 500 bp were removed (Hu et al., 2024). MetaGeneMark 2.10 was then used for open reading fragment (ORF) prediction (gene length >100 bp). CD-HIT 4.5.8 with parameters set at identity >95% and coverage >90% (-c 0.95, -G 0, -aS 0.9, -g 1, -d 0) was used to generate the initial non-redundant gene catalog (nrGC) (Fu et al., 2012). Subsequently, the clean reads were aligned to the nrGC using SOAPaligner 2.21 with parameters (-m 200, -x 400, identity ≥95%), and the ORFs abundance less than two were eliminated to create the unigenes (Perez-Prieto et al., 2024). A total of 289,983 scaftigs with an average length of 972.87 bps were obtained in the influent, which contained 462,594 ORFs. For the effluent, a total of 133,138 scaftigs with an average length of 1342.94 bps were obtained, containing 280,107 ORFs (Table 1).

**Table 1:** The assembly statistical information of each sample.

Sample source	Number of scaftigs	Total length (bp)	Largest length (bp)	Average length (bp)	N50 (bp)	N90 (bp)	GC (%)
Influent	289,983	282,114,566	39,003	972.87	990	556	51.07
Effluent	133,138	178,796,936	350,392	1342.94	1,611	593	54.39

### Bacterial taxonomy analysis, metagenome binning and phylogeny of metagenome-assembled genomes

For bacterial taxonomy analysis, DIAMOND 0.8.1 was employed to align unigenes against bacterial sequences extracted from the NCBI NR database using BLASTP with an E-value cutoff of 1 × 10^−5^ (Buchfink et al., 2015). For each unigene, the significant matches which were defined by *E*-values <10 × *E*-value of the top hit, were retained for taxonomic classification using lowest common ancestor (LCA) algorithm (Qin et al., 2010). Finally, sunburst illustration of relative abundance was performed to visualize bacterial taxonomy composition.

Metagenome-assembled genomes (MAGs) were assembled by metaWRAP-Binning module with three binning methods metaBAT2, MaxBin2 and CONCOCT, and the other parameters were set at default (Uritskiy et al., 2018). The MAGs were retained with completion >50, and contamination <10 (-c 50 -x 10). The taxonomy of recovered MAGs was determined by GTDB-Tk classify_wf. Phylogenetic analysis of these MAGs was performed using Fasttree, based on the 120 ubiquitous bacterial genes from GTDB (Chaumeil et al., 2019). And the tree was illustrated and annotated by iTol.^1^

### Detection of antimicrobial resistance genes, mobile gene elements, and plasmids

The ARGs were identified by BLAST (*E*-value <1 × 10^−4^) against the National Database of Antibiotic-Resistant Organisms (NDARO), with amino acid identity >90% and coverage >60% (Lan et al., 2023). “Last-resort” antibiotics including tigecycline, colistin, daptomycin, vancomycin, and linezolid are the last line of defence against antibiotic resistant pathogen infections (Li et al., 2022). A total of 26,065 and 7,819 16S rRNA reads were detected in the influent and effluent, respectively, according to the analysis using ARGs-OAP v3.2.4 (Li et al., 2024). The relative abundance of ARGs was calculated by normalizing read abundance against the length of each gene and 16S rRNA. Then, the maximum likelihood (ML) phylogenetic trees of the beta-lactam and aminoglycoside resistance genes were constructed by CLC genomics workbench.

Scaftigs containing ARGs were extracted with parameters of identity >90% and coverage >40%. The origin of these scaftigs, whether plasmid- or chromosome-derived, was predicted using RFPlasmid, which offers high specificity and sensitivity (up to 99%) based on short-read assemblies (van der Graaf-van Bloois et al., 2021). Insertion sequences (IS), integrons, and transposons are MGEs found in bacteria, and canonical MGEs (IS, integrons, and transposons) within 10-kb of the flanking sequence of ARGs were detected using BacAnt with identity >90% and coverage >40% (Hua et al., 2021). The connection network among ARGs, plasmids, and MGEs was visualized using Cytoscape v3.10.3.

### Statistical analysis

The relative abundance of ARGs was log-transformed and tested for normality using the Shapiro–Wilk test. The difference in relative abundance of ARGs between influent and effluent was then analyzed using non-parametric paired test (Wilcoxon matched-pairs test). The changes in relative abundance of ARGs in relation to their locations or adjacent MGEs, were analyzed by Fisher’s exact test. The statistical analyses were performed using R 4.4.1, and a *p*-value <0.05 was considered statistically significant.

## Results

### Bacterial community composition variation in hospital wastewater

**Figure 1:**
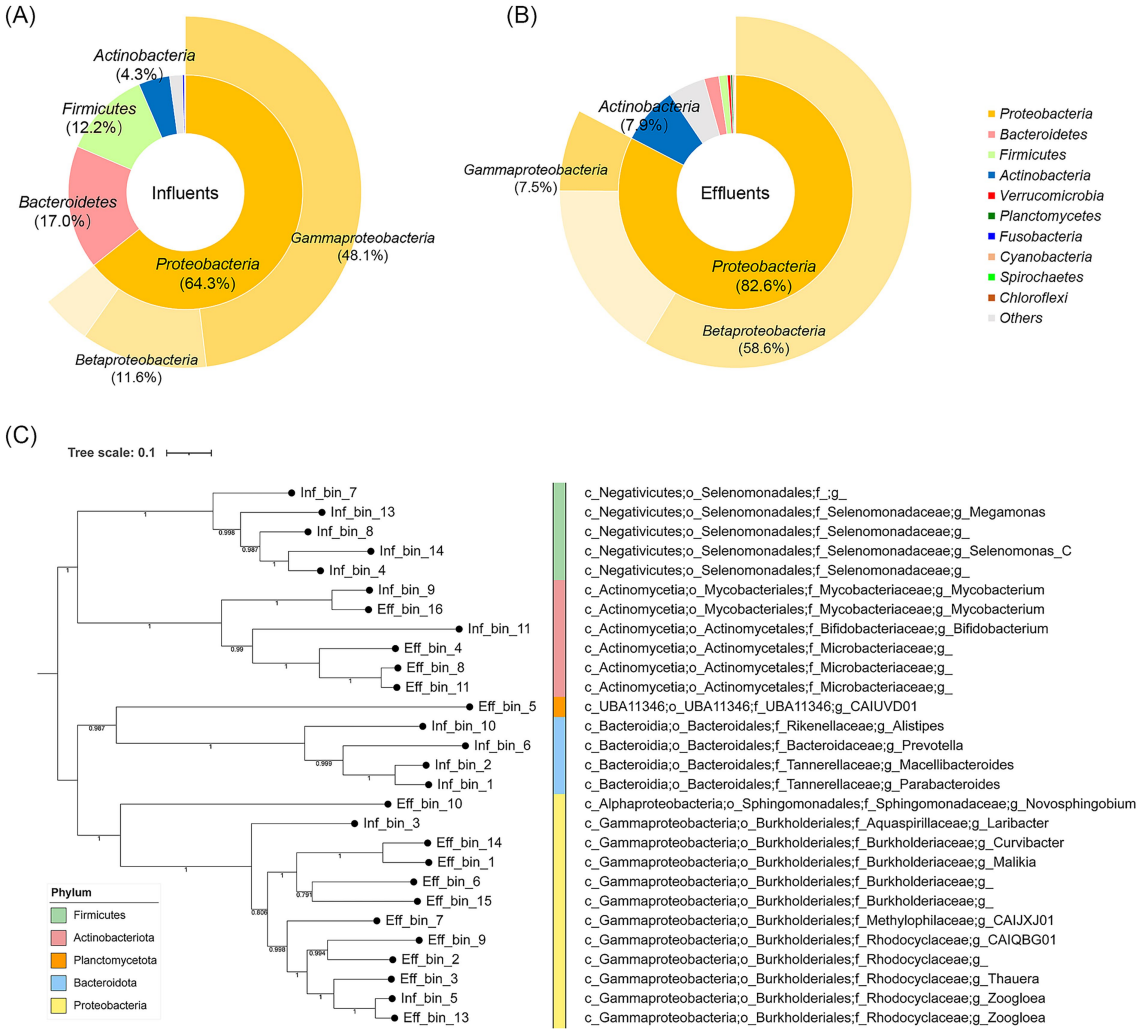
Bacterial composition in the influent **(A)** and the effluent **(B)** of hospital wastewater. The inner circle represents classification by phylum, and the outer circle represents classification by class. **(C)** Phylogenetic tree of MAGs in the influent and effluent.

To examine the distribution of bacteria in the HWW, the bacterial taxonomy analysis was performed based on scaftigs (Figures 1A,B). The results showed that *Proteobacteria* had the highest abundance at the phylum level in the influent and effluent, with a relative percentage of 64.3 and 82.6%, respectively. *Bacteroidetes* represented the second most abundant phylum in the influent (17.0%), while *Actinobacteria* ranked second in the effluent (7.9%). Within the *Proteobacteria* phylum, *Gammaproteobacteria* were the predominant class in the influent (74.8%), however, *Betaproteobacteria* were the most prevalent class in the effluent (70.9%), which indicated that the HWTS could effectively change the bacterial composition.

Then, to evaluate the bacterial residue after wastewater treatment, we performed phylogenetic analysis of MAGs (Figure 1C). We identified 28 qualified MAGs, including 13 MAGs from the influent, and 15 MAGs from the effluent, which belonged to five phyla (Supplementary Table S3). The results showed that some bacteria from the effluent were genetically closely related to those in the influent. For example, Inf_bin_9 from the influent and Eff_bin_16 from the effluent were closely related, and both belonged to *Mycobacterium* at the genus level. Inf_bin_5 from the influent and Eff_bin_13 from the effluent were closely related, and both were classified as *Zoogloea* at the genus level. These results indicated that some residual bacteria could not be eliminated by HWTS.

### Identification of ARGs in hospital wastewater

**Figure 2:**
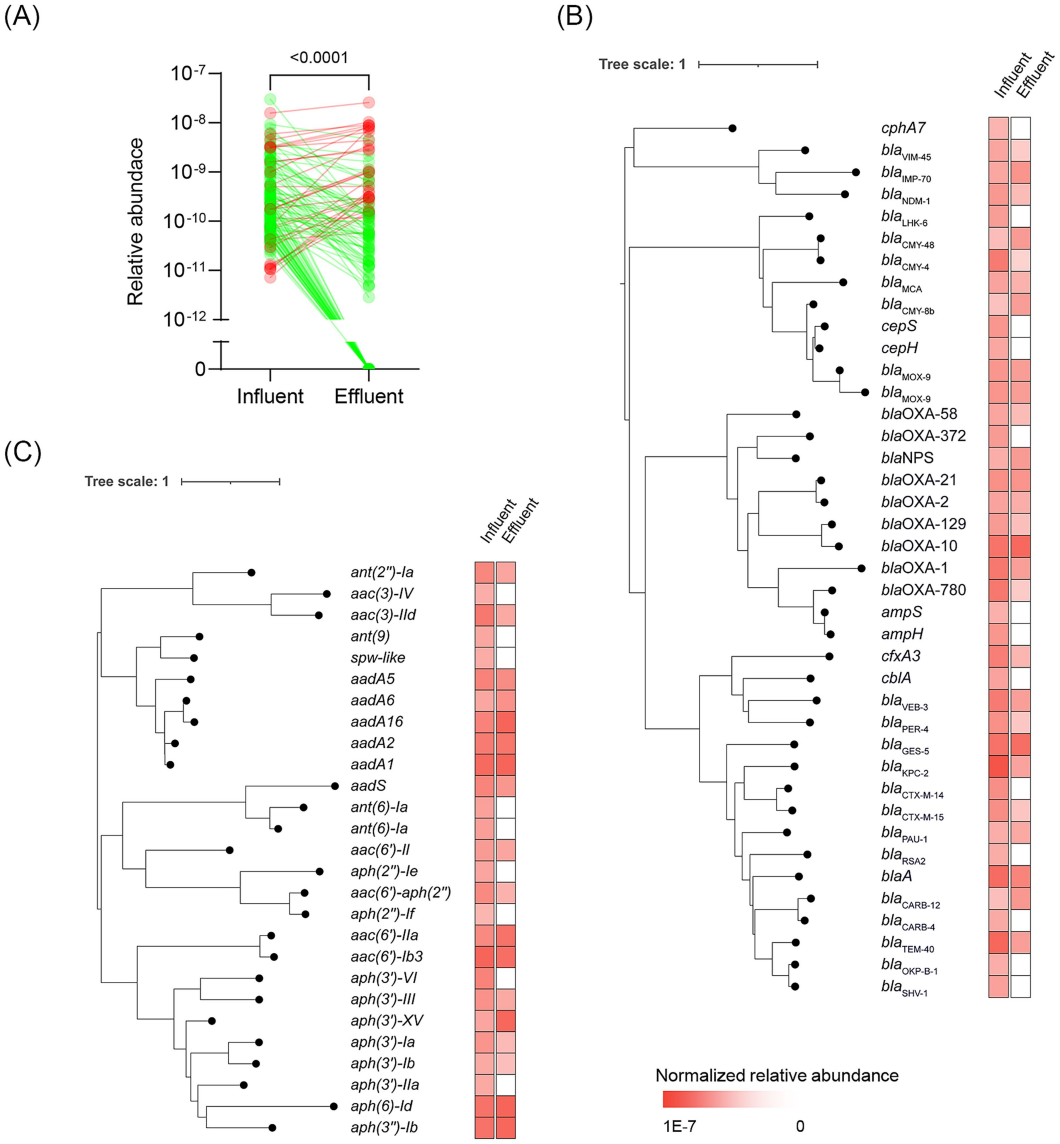
**(A)** Changes in the relative abundance of ARGs after wastewater treatment. Phylogenetic relationship of beta-lactam **(B)** and aminoglycoside **(C)** resistance genes in the HWW. The relative abundance of each gene was illustrated on the right.

We identified 140 non-redundant ARGs from both samples (Supplementary Table S4), with genes conferring beta-lactam resistance being the most prevalent (40/140). These beta-lactam resistance genes were further subdivided into those encoding extended-spectrum beta-lactamases (*n* = 5), such as *bla*_CTX-M_, *bla*_PER_, and *cfxA3*; and carbapenemases (*n* = 9), such as *bla*_KPC_, *bla*_VIM_, *bla*_NDM_, and *bla*_IMP_. Genes conferring aminoglycoside resistance were the second prevalent ARGs in the HWW (27/140), followed by genes conferring resistance to macrolides, phenicols, tetracyclines, and others. Then, we calculated the normalized relative abundance of these ARGs in both samples, and the results showed that the relative abundance of ARGs in the effluent was significantly lower than those in the influent (Figure 2A, *p* < 0.0001). Among these ARGs, 77.9% (109/140) decreased in the effluent compared to the influent, with 35.0% (49/140) undetectable in the effluent, indicating that the HWTS effectively reduced the prevalence of ARGs in the discharged wastewater (Supplementary Table S5). Moreover, the genes conferring resistance to beta-lactam, macrolide, and tetracycline significantly decreased in the effluent (*p* < 0.05, Supplementary Figure S1).

Among these ARGs, we identified 10 genes related to the “last-resort” antibiotic resistance. For example, vancomycin resistance genes, including *vanH-M*, *vanX-M*, and *vanM*, were identified, with *vanM* being considered a newly acquired glycopeptide resistance gene. Linezolid resistance genes *cfr(C)* and *optrA*, colistin resistance genes *mcr-4.3* and *mcr-5.1*, and tigecycline resistance genes *toprJ*, *toprJ1*, and *tet(X2)* were also identified. The normalized relative abundance of these genes significantly decreased in the effluent (Supplementary Figure S1, *p* = 0.002), and seven genes (7/10) could not be detected in the effluent, indicating that the HWTS could reduce the contamination of genes in association with “last-resort” antibiotic resistance. Notably, the relative abundance of two quaternary ammonium compound resistance genes, *qacF* and *qacG2*, increased in the effluent, suggesting that bacteria harboring these genes may survive the treatment process (Table 2).

**Table 2:** The relative abundance of genes conferring “last-resort” antibiotic resistance and *qac* genes in the influent and effluent.

ARGs	Class	Relative abundance		Changes in the effluent
		Influent	Effluent	
Genes conferring “last-resort” antibiotic resistance				
*vanH-M*	Glycopeptide	7.63 × 10^−11^	0	Decreased
*vanX-M*	Glycopeptide	8.09 × 10^−11^	0	Decreased
*vanM*	Glycopeptide	8.52 × 10^−11^	0	Decreased
*cfr(C)*	Lincosamide/macrolide/streptogramin	1.34 × 10^−10^	0	Decreased
*optrA*	Phenicol/oxazolidinone	6.02 × 10^−11^	0	Decreased
*mcr-4.3*	Colistin	8.40 × 10^−11^	0	Decreased
*mcr-5.1*	Colistin	3.91 × 10^−10^	1.16 × 10^−11^	Decreased
*toprJ*	Tetracycline	8.17 × 10^−11^	0	Decreased
*toprJ1*	Tetracycline	2.87 × 10^−09^	3.56 × 10^−11^	Decreased
*tet(X2)*	Tetracycline	3.99 × 10^−10^	1.39 × 10^−10^	Decreased
Quaternary ammonium compound resistance genes				
*qacF*	Quaternary ammonium	1.77 × 10^−10^	3.06 × 10^−10^	Increased
*qacL*	Quaternary ammonium	2.13 × 10^−10^	4.78 × 10^−11^	Decreased
*qacG2*	Quaternary ammonium	1.74 × 10^−10^	9.73 × 10^−10^	Increased

### Phylogenetic relationship of beta-lactams and aminoglycosides resistance genes in hospital wastewater

Since the beta-lactam and aminoglycoside resistance genes were prevalent, we performed the phylogenetic analysis of them (Figures 2B,C). The result showed that all the beta-lactams and aminoglycosides resistance genes could be found in the influent, however, 22.5% (9/40) of beta-lactam resistance genes and 48.1% (13/27) of aminoglycosides resistance genes were undetectable in the effluent. We also found some ARGs exhibited an elevated relative abundance in the effluent, such as *aadA* and *bla*_OXA-10_, which indicated that these ARGs were persistent in the sewage.

### Identification of ARG locations and their linkage with mobile genetic elements

**Figure 3:**
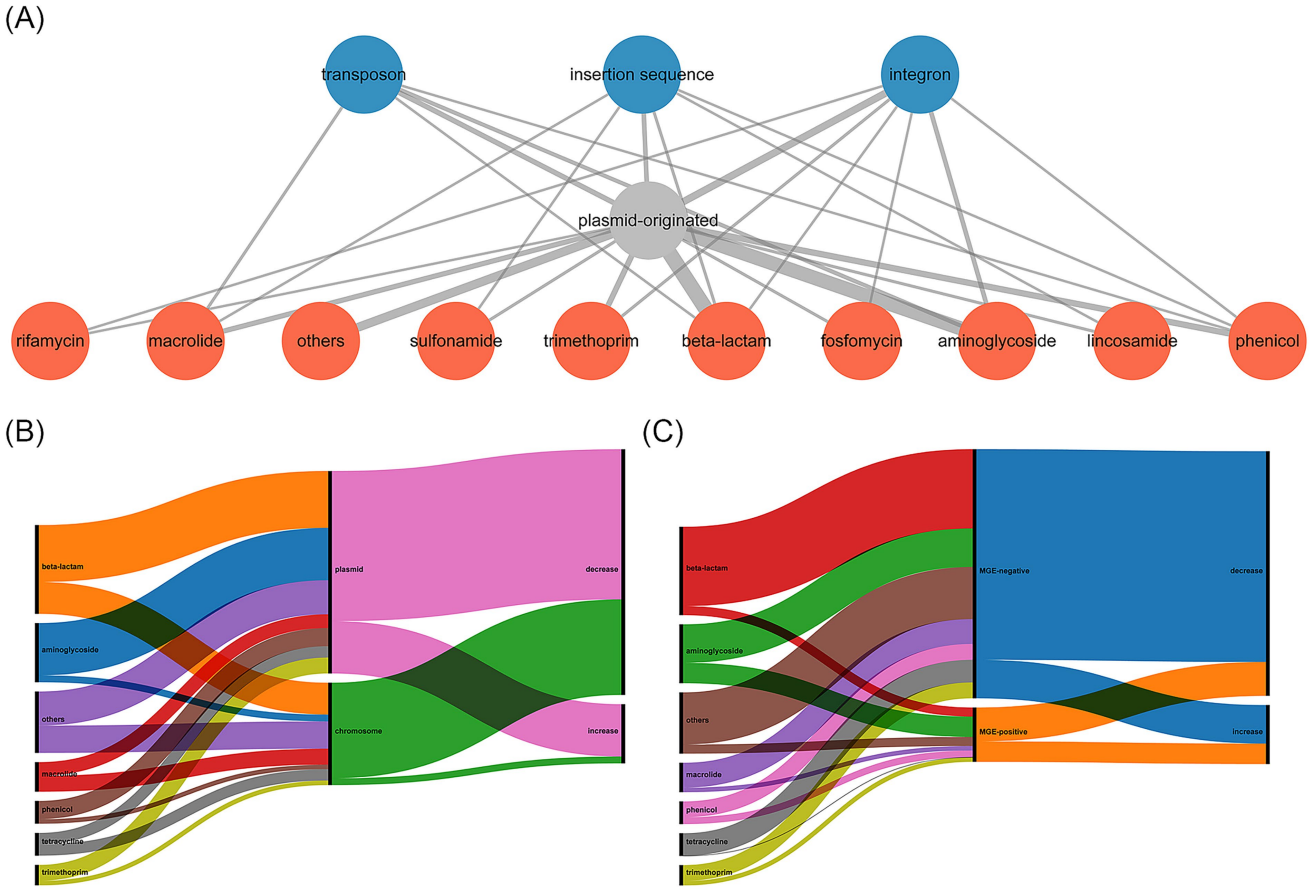
**(A)** Relationship among AGRs, ARGs locations, and ARGs surrounding environments. Red nodes represent the resistance gene types, grey node represents ARGs were located on plasmids, and blue nodes represent ARGs were adjacent to MGEs. The thickness of edge represents the number of interactions between nodes. **(B)** Distribution and linkages among ARGs, ARGs locations, and relative abundance changes in the effluent. **(C)** Distribution and linkages among ARGs, ARGs surrounding environments, and relative abundance changes in the effluent.

To understand the potential mobility of ARGs, we analyzed the origin of ARG-harboring scaftigs, and identified adjacent MGEs (Table 3, Figure 3A and Supplementary Table S6). We found that 95.7% (134/140) of the ARGs had the identical origins in both samples. By excluding inconsistent results, the results showed that 66.4% (89/134) of ARGs were located on plasmids, and the rest were located on chromosomes. Moreover, we compared changes in the relative abundance of ARGs based on their genomic location. The results showed that ARGs located on plasmids exhibited a significantly smaller reduction in relative abundance than those located on chromosomes (Figure 3B and Supplementary Table S7, *p* = 0.01). In addition, 17.9% (24/134) of ARGs were adjacent to MGEs, encompassing nine classes of ARGs, and the majority (21/24) of these MGEs were associated with plasmid-derived scaftigs. We compared changes in the relative abundance of ARGs based on their surrounding genetic environments. The results showed that ARGs adjacent to MGEs exhibited a significantly smaller reduction in relative abundance than those not adjacent to MGEs (Figure 3C and Supplementary Table S8, *p* = 0.02). These results revealed that the ARGs originating from plasmids or adjacent to MGEs were negatively correlated with the reduction in relative abundance after HWTS treatment.

**Table 3:** The detailed connection between ARGs and MGEs.

ARGs	Class	Origin of scaftigs	MGEs
*bla*_KPC-2_	Beta-lactam	Plasmid	IS: IS*Kpn6*
*bla*_IMP-70_	Beta-lactam	Plasmid	Integron: In*829*
*bla*_CTX-M-14_	Beta-lactam	Plasmid	Transposon: Tn*602*
*bla*_OXA-780_	Beta-lactam	Chromosome	IS: IS*1247*
*aph(3′)-Ia*	Aminoglycoside	Plasmid	Transposon: Tn*4352*
*aph(3″)-Ib*	Aminoglycoside	Plasmid	Transposon: Tn*5393*, Tn*6205*
*aac(6′)-aph(2″)*	Aminoglycoside	Plasmid	Transposon: Tn*4401*
*aac(6′)-Il*	Aminoglycoside	Plasmid	Integron: In*547*
*aadA5*	Aminoglycoside	Plasmid	Integron: In*139*
*aph(6)-Id*	Aminoglycoside	Plasmid	Transposon: Tn*5393*, Tn*6205*
*aadA16*	Aminoglycoside	Plasmid	Integron: In*1021*
*aph(3′)-XV*	Aminoglycoside	Plasmid	Integron: In*1021*
*aac(6′)-IIa*	Aminoglycoside	Plasmid	Integron: In*231*
*lnu(C)*	Lincosamide	Plasmid	IS: IS*Sag10*
*erm(F)*	Macrolide	Chromosome	Transposon: Tn*4551*
*mph(A)*	Macrolide	Plasmid	IS: IS*26*, Transposon: Tn*6292*
*fosE*	Fosfomycin	Chromosome	Integron: In*689*
*catA1*	Phenicol	Plasmid	Transposon: Tn*9*-like
*catB8*	Phenicol	Plasmid	Integron: In*90*
*cmx*	Phenicol	Plasmid	IS: IS*5564*
*arr-3*	Rifamycin	Plasmid	Integron: In*63*
*dfrA14*	Trimethoprim	Plasmid	Integron: In*90*
*dfrA27*	Trimethoprim	Plasmid	Integron: In*90*
*sul2*	Sulfonamide	Plasmid	IS: IS*Vsa3*

## Discussion

This study has some limitations that should be considered when interpreting the results. First, the samples were collected at a single time point, which may not accurately represent the environmental effects at the site of collection. Second, culture-based methods were not used to screen for bacteria harboring specific ARGs, making it impossible to determine whether the ARGs were carried by bacteria or existed extracellularly.

Asfaw et al. (2017) has isolated numerous ARB species of clinical relevance in HWW, such as *Klebsiella* spp., *Staphylococcus aureus*, *Pseudomonas aeruginosa*, and *Salmonella* spp., and it was found that the HWTS could reduce bacterial counts by more than 10^4^-fold. Limayem et al. (2019) determined that the proportion of *Gammaproteobacteria* decreased from 36.18 to 3.17% after wastewater treatment, highlighting the important role of HWTS in reducing the dissemination of potential pathogens, as many human bacterial pathogens belong to *Gammaproteobacteria* class. MAG analysis revealed the presence of strains with a close relationship between influent and effluent, such as *Mycobacterium* and *Zoogloea*, suggesting that these isolates might persist through treatment.

Moreover, HWW serves as a hotspot reservoir of ARGs, and previous studies have shown that the profiles of ARGs in HWW were highly diverse (Huang et al., 2023). For example, Yao et al. (2021) revealed that *bla*_GES-1_ had the highest relative abundance among beta-lactam resistance genes. In the current study, we found that *bla*_KPC-2_ exhibited the highest relative abundance among the beta-lactam resistance genes in the HWW. Plasmids and MGEs play crucial roles in the spread of ARGs, facilitating the rapid dissemination of resistance determinants among bacteria (Peterson and Kaur, 2018). Our finding indicated that 66.4% of ARGs were located on plasmids, and 17.9% were adjacent to MGEs, suggesting the potential mobility of these ARGs. We found 80.0% (4/5) of genes encoding extended-spectrum beta-lactamases were located on plasmids, with *bla*_CTX-M-14_ associated with Tn*602*. Additionally, 66.7% (6/9) of carbapenemase genes were located on plasmids, with *bla*_KPC-2_ adjacent to IS*Kpn6* and *bla*_IMP_ located in integron In*829*, a core structure commonly reported in previous studies (Zhao and Hu, 2011; Zhang et al., 2017; Dong et al., 2018). The increased ARGs, along with the persistent strains, warrant further investigation. Notably, previous studies have shown that water chlorination could facilitate the transformation and conjugation of plasmid-encoded ARGs, thereby elevating ARG abundance (Zhang et al., 2021).

According to national standards, hospital wastewater treatment in China is generally carried out using a three-stage treatment process. In the HWTS investigated in this study, the primary treatment method is biological contact oxidation, while other methods, such as the activated sludge process and membrane bioreactor, are also commonly employed in hospital wastewater treatment. They had a similar biological treatment based on biodegrading of pollutants through bacterial respiration (Majumder et al., 2021). The bio-oxidation process leads to antibiotic-resistant bacteria (ARB) cell lysis and partially degrades their genetic material. Furthermore, the released extracellular DNA can adsorb onto sludge or sediment (Zhang et al., 2024). However, none of these methods have a mechanism specifically designed to remove ARGs. Zhu et al. (2023) found that certain types of ARGs, such as beta-lactam and aminoglycoside resistance genes, were more abundant in effluent, indicating these ARGs could not be significantly removed by chlorination. Thus, a lot of novel methods have been developed to remove ARGs in the wastewater. Hsiao et al. (2023) reported that ozone micron bubble pretreatment showed superior efficiency in ARGs degradation, which could reduce the abundance of *sul1*, *tetA*, *bla*_TEM-1_, and *mcr-1* in 20 min reaction time. Zhang et al. (2016) reported that advanced oxidation process including Fenton oxidation and UV/H_2_O_2_ process could reduce ARGs effectively. However, novel hospital wastewater treatment technologies are still in the developmental stage, and most existing studies have been conducted at the lab scale using synthetic wastewater.

## Conclusion

This study provided an evaluation of the impact of HWTS, which changed the bacterial composition, reduced ARGs abundance, and notably blocked the spread of the genes conferring “last-resort” antibiotics resistance. Future studies should focus on active monitoring of HWW effluents and the development of improved HWW treatment processes.

## Data Availability

The datasets presented in this study can be found in online repositories. The names of the repository/repositories and accession number(s) can be found below: https://www.ncbi.nlm.nih.gov/, PRJNA1087003.

## Footnotes

^1^ https://itol.embl.de/
