# Supplementary material for: Evaluating the effects of hospital wastewater treatment on bacterial composition and antimicrobial resistome
Source: Front Microbiol. 2025 Oct 2;16:1620677. doi: 10.3389/fmicb.2025.1620677 (PMC12529969; doi:10.3389/fmicb.2025.1620677)
Supplement: Supplementary file 1 [file Table_1.DOCX]

Figure S1 Changes in the relative abundance of different ARGs types after wastewater treatment.
